# GAM: General Auxetic Metamaterial with Tunable 3D Auxetic Behavior Using the Same Unit Cell Boundary Connectivity

**DOI:** 10.3390/ma16093473

**Published:** 2023-04-29

**Authors:** Ismael Ben-Yelun, Guillermo Gómez-Carano, Francisco J. San Millán, Miguel Ángel Sanz, Francisco Javier Montáns, Luis Saucedo-Mora

**Affiliations:** 1 Escuela Técnica Superior de Ingeniería Aeronáutica y del Espacio, Universidad Politécnica de Madrid, Pza. Cardenal Cisneros 3, 28040 Madrid, Spain; i.binsenser@upm.es (I.B.-Y.); guillermo.gocarano@alumnos.upm.es (G.G.-C.); sanmillan@inta.es (F.J.S.M.); miguelangel.sanz@upm.es (M.Á.S.);; 2 Instituto Nacional de Técnica Aeroespacial Esteban Terradas, Carretera de Aljavir, Km 4, 28850 Torrejón de Ardoz, Spain; 3 Department of Mechanical and Aerospace Engineering, Herbert Wertheim College of Engineering, University of Florida, Gainesville, FL 32611, USA; 4 Department of Materials, University of Oxford, Parks Road, Oxford OX1 3PJ, UK; 5 Department of Nuclear Science and Engineering, Massachusetts Institute of Technology, Cambridge, MA 02139, USA

**Keywords:** metamaterial, auxetic, variable properties, mimetic

## Abstract

Research on auxetic metamaterials is important due to their high performance against impact loadings and their usefulness in actuators, among other applications. These metamaterials offer a negative Poisson’s ratio at the macro level. However, usual auxetic metamaterials face challenges in (1) grading the effect, (2) coupling and combining auxetic metamaterials with non-auxetic materials due to boundary compatibility, (3) obtaining the same auxetic behavior in all directions in the transverse plane, and (4) adapting the regular geometry to the component design boundary and shape. The goal of this paper is to present a novel, recently patented tunable 3D metamaterial created to reproduce a wide spectrum of 3D auxetic and non-auxetic Poisson’s ratios and Young’s moduli. This wide range is obtained using the same basic unit cell geometry and boundary connections with neighboring cells, facilitating designs using functionally graded metamaterials as only the connectivity and position of the cell’s internal nodes are modified. Based on simple spatial triangularization, the metamaterial is easily scalable and better accommodates spatial curvatures or boundaries by changing the locations of nodes and lengths of bars.

## 1. Introduction

The use of metamaterials dates back to World War II [1,2], but has recently gained renewed interest with the increased use of 3D printing techniques, which eliminate most of the shape constraints due to manufacturing procedures [3]. This is particularly important when addressing structures at different scales, creating true multiscale architected patterns with salient mechanical properties typical of metamaterials, which is the main difference from classical manufacturing processes [4,5]. Currently, this technique is mainly used for small batches and value-added components due to speed and cost, but with continuous improvements in 3D printing technology, the approach is highly promising even for mass production industries [6,7]. According to Fortune Business Insights [8], the 3D printing market is expected to grow at a rate of 24.3% in the next few years or about nine times in the next decade. The main potential of 3D printing lies in the tailored design and detailed multiscale manufacturing allowed by the technique, which enables the metamaterial structure to span the material design space. At a larger scale, metamaterials can be considered as continuum material [9,10], since in essence, all materials have microstructures.

There is no unique definition of what a (mechanical) metamaterial is; some authors tie the meaning to an architected structure that brings unique (i.e., not found in nature) physical properties. Other authors think of metamaterials as architected structures at the micro level, which bring a unique combination of properties (e.g., mechanical) at the continuum level [11,12] (p. 3), among many others. For a more detailed review on metamaterials, the reader is referred to Bertoldi et al. [13] and Barchiesi et al. [14].

There is a large family of metamaterials; however, the present work focuses on auxetic mechanical metamaterials. The term auxetic was coined in 1991 by Evans [15], and the possibility of materials presenting a negative Poisson’s Ratio (NPR) was considered by Love a century ago [16]. Relevant works about NPR materials were published by Almgren [17] and Wojciechowski [18] more than three decades ago. Recent studies about this effect in other materials, e.g., graphene, can be found in [19]. A general classification of auxetic materials can be found in [20,21], and for a deeper review of auxetic materials, the reader is referred to [22,23,24,25].

Regarding auxetic metamaterials, these are functional microstructures that, at the continuum level, result in a negative Poisson’s ratio, at least in one direction. These metamaterials have potential applications in structures under impact loadings, among many other applications [26,27,28,29,30]. Of course, auxetic materials are not necessarily metamaterials. Auxetic foams are also typical [31,32,33].

Usual auxetic metamaterials present some practical inconveniences when designing components using such materials. The first is that the auxetic properties may only be desirable in some parts of the component. The second is that typical metamaterials struggle to accommodate different sizes and shapes, including curvature and boundary conditions, while maintaining good connectivity. This limits some of the advantages of 3D printing in shape designs. One challenge in functional metamaterials is to link them with other functionally graded metamaterials or conventional metamaterials, as different metamaterial cells typically result in different boundaries and, therefore, different connecting nodes or surfaces. The mixture of graded auxetic metamaterials and auxetic with non-auxetic metamaterials presents significant challenges due to strain inhomogeneity in connecting zones [34,35].

Many metamaterial designs have tunable auxetic behaviors, most in 2D, but some in 3D; see, e.g., [36,37,38,39] for more details. The salient feature of this metamaterial is that it allows for a wide range of auxetic or conventional behaviors. The structure is three-dimensional, and changes in the topology of the metamaterial (to provide different behaviors) are minimal, involving mere position relocations of some nodes or changes in internal connectivities. Additionally, the auxetic behavior in the transverse plane is orthotropic and can be close to the transverse isotropic, if desired. The metamaterial presented in this paper allows for a smooth (or abrupt) transition between the auxetic and non-auxetic configurations, and even different levels of auxeticity, as both behaviors can be obtained and tailored using the same unit cell size, overall shape, and the same boundary connectivity. Only a change in the connectivity or the positions of internal lattices is needed to tailor its behavior. Moreover, the 3D triangularization and overlapped cell volumes allow for simpler accommodation of curvatures and boundaries, typically needed in component design, by simply changing nodal locations and bar lengths (note that the simplest mesh generators to adapt volumes are based on triangularization). The type of triangularization used in our metamaterial results in an in-plane hexagonal honeycomb-like structure, making the resulting metamaterial quasi-transversely isotropic, in contrast to typically markedly orthotropic metamaterials. Notably, unlike other recent works [40,41], the change from auxetic to conventional behavior is not pursued through the deformation of the metamaterial, but rather through a versatile connectable family of undeformed cell designs.

The goal of this paper is to present the General Auxetic Metamaterial (GAM), a structure with tunable macroscopic properties, whose unit cell is registered under patent reference ES2907514 A1 [42]. This patent has novelty against inventions in the INVENES (Spanish) [43] and EPODOC (EPO worldwide bibliographic data) [44] databases. While some patents describe auxetic metamaterial configurations, none present tunable properties similar to the invention in this paper [45,46]. The presented metamaterial allows for the spatial changes of mechanical properties by varying relative cell dimensions, lattice radii, volume fractions, or curvatures [47,48,49,50], which are the primary techniques used for the graded variation of mechanical properties in metamaterials. In addition, the proposed unit cell can change the Young’s modulus and Poisson’s ratio through different connectivity configurations while keeping the same radius and dimensions of the cell and the same number of lattices, maintaining more constant density and porosity properties.

Section 2 is dedicated to the geometrical description of the unit cell and how the auxetic and non-auxetic effects are obtained. Then, we show how the properties of the cell allow for a very simple methodology to create shell structures with this metamaterial. Moreover, given the simple geometric variables defining the cell and the resulting behavior, we demonstrate that a machine learning algorithm can be developed to link the set of geometric features and desired output. These techniques can be used to perform straightforward nonlinear inverse analyses to obtain the metamaterial layout for specific mechanical requirements.

## 2. Theoretical Description of the Unit Cell

**Figure 1:**
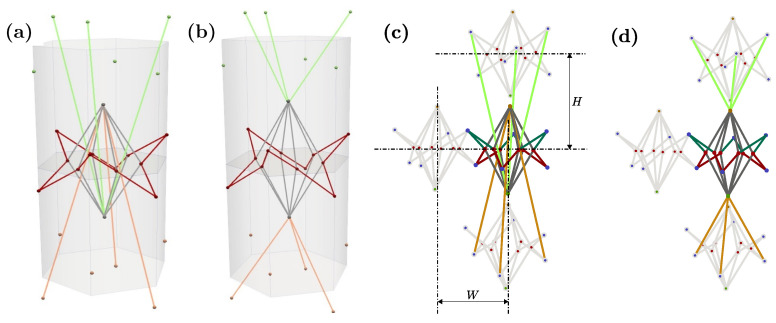
The unit cell in auxetic configuration (**a**), the non-auxetic configuration (**b**), the connection between the auxetic unit cell with its neighbors (**c**), and the connection between the conventional unit cell with its neighbors (**d**).

As mentioned earlier, the GAM is designed to reproduce a wide range of auxetic/non-auxetic behaviors by changing the geometrical configuration of its unit cell. Specifically, the unit cell is a lattice structure comprised of connected struts at given joints, or nodes. Therefore, modifying the position of these nodes leads to different macroscale behaviors, while the global shape of the unit cell is preserved. This means that cells with different auxetic properties can be combined to create graded variations of the mechanical properties in the resulting component. This behavior is studied when the resulting material is loaded and aligned with the height of the prism in the unit cells. Figure 1 shows the auxetic (a) and non-auxetic (b) configurations, as well as the link between unit cells (c), both vertically and horizontally.

**Figure 2:**
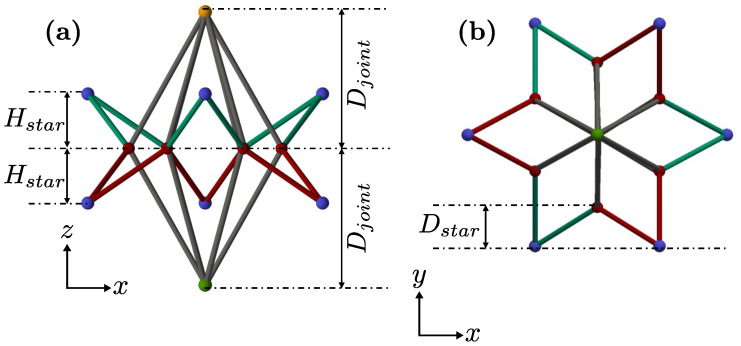
Lateral (**a**) and top (**b**) views of the unit cell and its key dimensions.

Additionally, Figure 2 shows that the height and position of the blue nodes are defined by the shape parameters of the unit cell, namely $D_{star}$ (see Figure 2b), which represents the penetration of the star within the apothem of the hexagon, and $H_{star}$, which represents the height of the external nodes. Finally, the height of the external nodes (in orange and green in Figure 2) is defined as $D_{joint}$.

In Figure 2b, a 12-sided polygon is displayed in the plan view. It should be noted that other configurations are also possible, such as one with a concave 8-sided octagon plan view or one with a concave 16-sided polygon plan view.

The main factor determining the switch between the auxetic and non-auxetic response is how the unit cells are stacked vertically, which is indicated by the green and orange bars in Figure 1. These bars are respectively connected to the nodes above and below the mean transverse plane (MTP) of their vertically adjacent cells, and vice versa.

**Figure 3:**
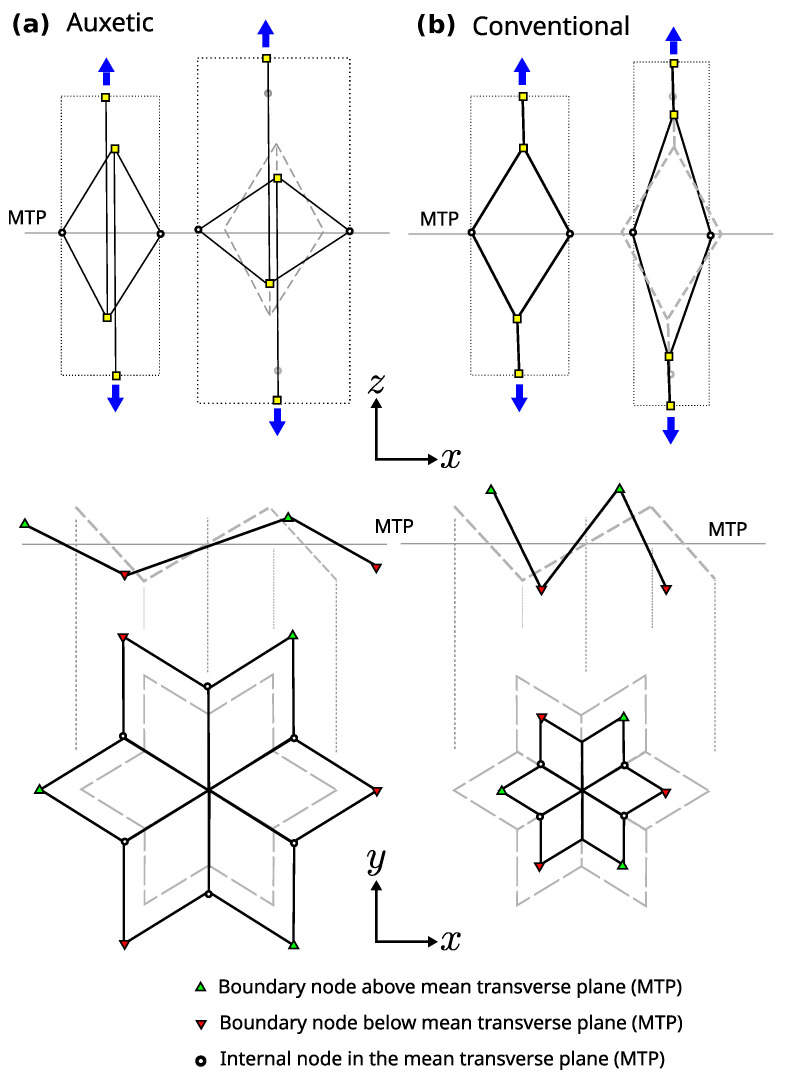
Auxetic (**a**) and conventional (**b**) lateral and top (plan) schematic views of the unit cells in different configurations.

The geometric ideas that give rise to the desired properties of GAM are depicted in Figure 3. The metamaterial is quasi-transversely isotropic, with a mean transverse plane (MTP in Figure 3) where most of the internal nodes are located. The top drawings of Figure 3 illustrate how the auxetic or conventional behavior is achieved by actions perpendicular to the MTP. These actions cause the folding or unfolding of the 3D star depicted in the bottom elevation-and-plan drawings of that star in Figure 3 (note that it is a 3D structure with nodes above and below the MTP, which facilitates folding and unfolding resulting in conventional and auxetic behavior). The connection of the basic parts shown in Figure 3 is provided in the metamaterial cell described in Figure 1. Note that, despite the explanation given to justify the geometry, the auxetic behavior is also achieved in the transverse direction if the metamaterial is loaded in the transverse plane (i.e., it is an auxetic metamaterial in 3D).

The green and orange bars in Figure 1 are responsible for changing the auxetic properties by linking to the top or bottom nodes of the neighboring unit cells (as also shown in Figure 3). The resulting Poisson’s ratio is calibrated by the position of the internal nodes. The evolving hexagonal prism, shown in light gray in Figure 1a,b, can be considered as the volume of the unit cell, with the centers of the top and bottom neighbors located at both ends of the prism. This means that, in contrast with classical metamaterials, there is no definite space dedicated only to one unit cell. In the vertical direction (Figure 1), cells are engaged in a less trivial way than in the typical metamaterial configuration, where each unit cell is typically an independent block without overlapping with its neighbors.

Parameters *W* and *H* (Figure 1c) define the height between layers as well as the horizontal sizes of the metamaterial. In terms of the evolving hexagonal prism, *H* is half of the height of the prism, and *W* is two times the apothem of the base. With these dimensions, the unit cells are defined, and the hexagons are configured as a honeycomb to engage with their neighbors in the layer. This is then replicated in space to create the different layers of the metamaterial.

In the auxetic configuration, the internal star will open when a vertical tension (i.e., loading along the *z*-axis according to Figure 1 and Figure 2) is applied, which is the load that will reduce the distance between the top and bottom nodes. This behavior will show a negative Poisson ratio.

The other configuration, shown in Figure 1b, exhibits non-auxetic behavior with a positive Poisson ratio. This case is achieved by configuring the star to open when the external nodes (top and bottom) are closer. This is accomplished by directly connecting the unit cells vertically (Figure 1b) instead of using cross-connections (Figure 1a).

**Figure 4:**
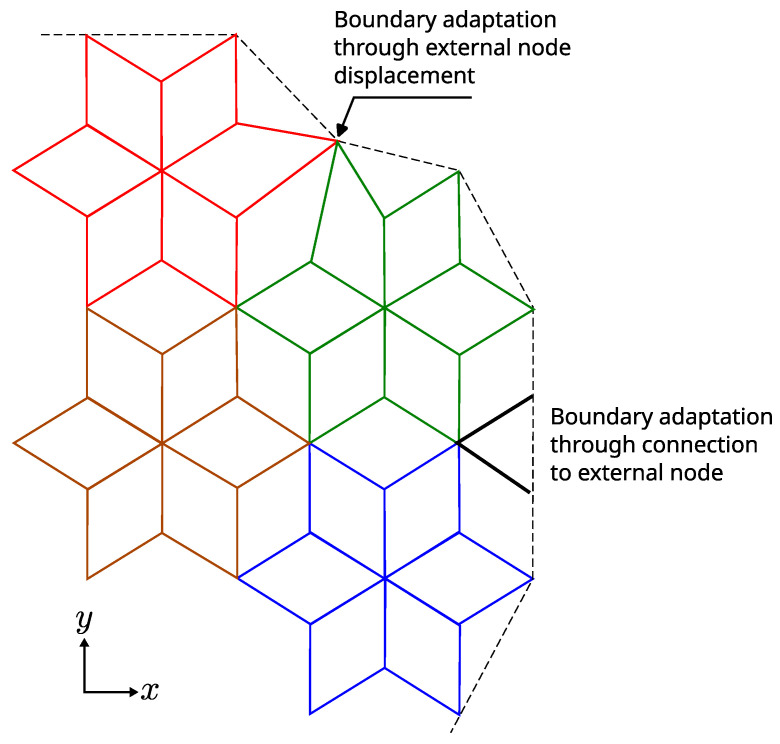
Simple solution to accommodate general boundaries through external nodes.

Finally, we note that the 3D triangular-based structure along the honeycomb plan allows for simple solutions to accommodate complex boundaries. Figure 4 shows some simple solutions to accommodate general boundaries through external nodes.

## 3. Examples of Simple Implementations of the Metamaterial

The objective of this section is to demonstrate that the proposed metamaterial can achieve a wide range of mechanical properties and be used in complex geometries using simple techniques. Therefore, this section does not present specific methodologies but focuses on the potential of the metamaterial. Firstly, we demonstrate that the metamaterial can be applied to construct components with different curvatures, such as fuselages. Secondly, we use an Euler–Bernoulli beam model with two nodes per beam and infinitesimal strains to model the metamaterial. The model shows that a wide spectrum of mechanical properties can be achieved by varying the geometrical variables. Finally, we demonstrate that the behavior of the metamaterial can be predicted using a simple machine learning model that acts as a high-dimensional nonlinear interpolation method. Even with a small training dataset, the model can accurately reproduce the performance of the metamaterial in an elastic compressive test.

### 3.1. Shell Generation with Layers of This Metamaterial

**Figure 5:**
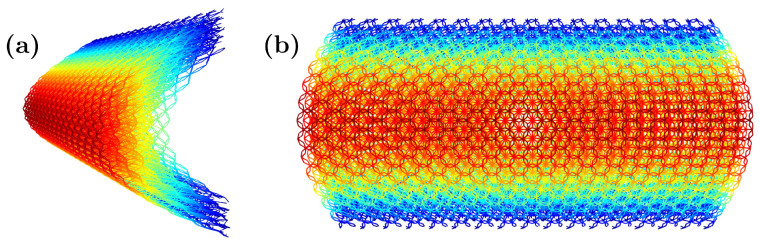
Three-dimensional (**a**) and top (**b**) views of a shell created from the proposed metamaterial cell simply using $\Delta z_{n}=20\;\sin\left(\frac{y_{n}}{10}\right)$.

Interestingly, the proposed metamaterial cell has the same dimensions and connectivity regardless of the intended mechanical behavior. Additionally, the cell is based on a simple spatial triangularization, which allows for any curvature without significantly changing the properties. As a result, a whole metamaterial shell can be easily generated by considering the shape and size of the unit cell, the number of layers, and the desired final size of the shell. Although the geometry is not as straightforward as other cells, such as the cubic cell, it is periodic and can be implemented and computed with versatility. By stacking the unit cell, a rectangular periodic lattice is formed, which can be mapped to the desired spatial shape. In the following, a couple of examples are displayed by adding to the *z*-coordinate of each node a mapping function $\Delta z_{n}=f\left(x_{n},y_{n}\right)$ for illustration purposes (of course, more complex transformations of the nodal coordinates are possible). The example in Figure 5 is calculated by simply using:

(1) $$\Delta z_{n}=20\;\sin\left(\frac{y_{n}}{10}\right)$$

to calculate the added $\Delta z_{n}$ to the $z_{n}$ coordinate for every point in a ($x_{n}$, $y_{n}$) location of the layer. Figure 5 shows different views of the sample calculated, which can be used, for example, to protect the edge of a blade or as a structure to guarantee the impact resistance (local and global through the shell effect).

**Figure 6:**
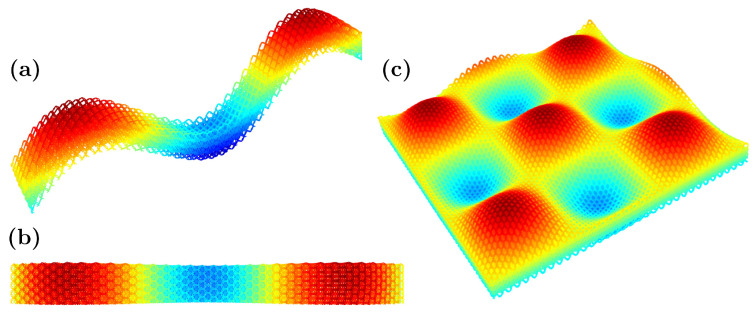
Different sections of a shell created from the proposed metamaterial cell using $\Delta z_{n}=10\;\sin\left(\frac{x_{n}}{10}\right)\;\sin\left(\frac{y_{n}}{10}\right)$, where (**a**) is a slice perpendicular to the *x*-axis, (**b**) is a top (plan) view of the previous slice from the *z*-axis, and (**c**) represents the whole domain.

For the different shell metamaterial structure given in Figure 6, we simply use

(2) $$\Delta z_{n}=10\;\sin\left(\frac{x_{n}}{10}\right)\;\sin\left(\frac{y_{n}}{10}\right).$$

The resulting shape of Figure 6 demonstrates that complex geometries and configurations, with tailored localized behaviors, may be obtained by simple transformations thanks to the salient properties and connectivity of the proposed metamaterial cell.

In the examples given in Figure 5 and Figure 6, the color scheme only shows the $z_{n}$ coordinates of the bars in each of the five layers implemented. The unit cell parameters used in the demonstrative examples of this section are $H=2\;\;\mathrm{mm},W=1\;\;\mathrm{mm},D_{joint}=-0.6\;\;\mathrm{mm}\;(\mathrm{auxetic}\;\mathrm{configuration}),D_{star}=0.2\;\;\mathrm{mm},$ $H_{star}=0.3\;\;\mathrm{mm}$. We also employed 5 layers through the thickness and a grid of 100×100 unit cells.

### 3.2. Numerical Testing of the Metamaterial

An Euler-Bernoulli beam model was used to test the different mechanical properties presented by the metamaterial with the different geometrical configurations. For this, the beam model with 6 degrees of freedom per node was composed of 5 vertical layers, and in each one, there was a 5×5 grid of unit cells. A detailed description of the structural matrix calculus, the Young’s modulus, and Poisson’s ratio calculation can be found in Appendix A. The model was loaded under compression by imposing a vertical displacement (Δu along the *z*-axis according to Figure 1 and Figure 2) on the top face nodes, and a vertical displacement restriction on the bottom face nodes. The base material parameters are shown in Table 1.

A total of 10,539 simulations were performed with different geometrical configurations of the key shape variables. These simulations resulted in both auxetic and non-auxetic behaviors with a wide range of Poisson’s ratios and stiffness values.

**Figure 7:**
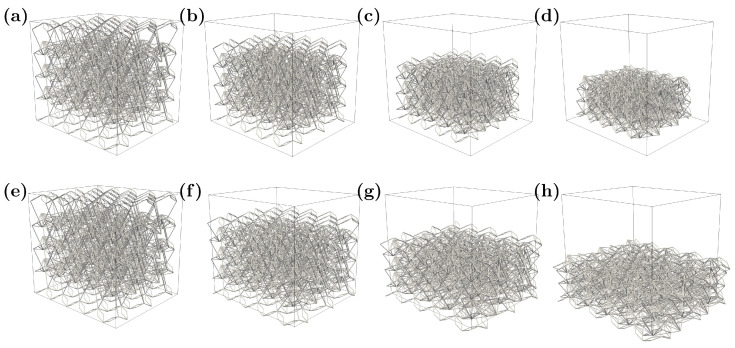
Typical numerical results for the auxetic (top, **a**–**d**), and non-auxetic (bottom, **e**–**h**) configurations. In both configurations, an increasing incremental vertical displacement load is applied—from unloaded (left) to fully loaded (right).

As demonstrative examples of such models, two configurations are shown here with the auxetic response in Figure 7a–d and the non-auxetic response in Figure 7e–h. The non-auxetic behavior is achieved by changing the sign of $D_{joint}$, which is equal to 0.6 mm in this case.

In Figure 7, each column represents a different load step. In the next subsection, a sensitivity analysis of the numerical results is presented to study the relationship between the mechanical properties related to the main direction and the geometrical variables presented in the previous sections of the paper.

**Figure 8:**
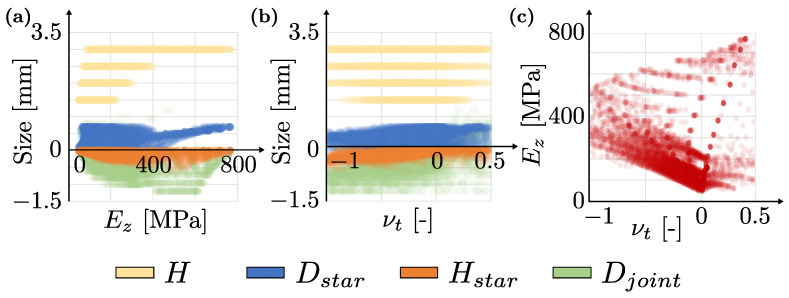
Range of mechanical properties obtained from the numerical models, considering different geometries, where (**a**,**b**) represent the magnitude (size) of these geometrical variables against the equivalent Young’s modulus $E_{z}$ and transverse Poisson’s ratio $\nu_{t}$, respectively, and (**c**) displays the relationship between Young’s modulus and Poisson’s ratio in the simulations.

For each set of geometric parameters, the vertical Young’s modulus and the transverse Poisson’s ratio are calculated. Figure 8 shows these mechanical properties plotted against the values of the unit cell’s geometric variables. These results may not be easy to interpret directly as a raw dataset. For this reason, the next subsection demonstrates (through a simple machine learning model) that those results can be easily interpreted.

Figure 8 shows the values of the different geometrical descriptors and their correlation with the mechanical properties, as well as the relationship between the Young’s modulus and the Poisson’s ratio of each calculation. $E_{z}$ is the macroscopic Young’s modulus in the loading direction (*z*-axis) of the uniaxial test from numerical simulation (Figure 7), and $\nu_{t}$ is the Poisson’s ratio relating to the transverse strain $\varepsilon_{t}$, with respect to the loading direction $\varepsilon_{zz}$, where the transverse strain is computed as

(3) $$\varepsilon_{t}=\frac{1}{2}\left(\varepsilon_{xx}+\varepsilon_{yy}\right).$$

The width *W* was considered constant and equal to 1 for all calculations. Table 2 shows the range and basic statistics of the geometrical and mechanical variables of this numerical model to provide an illustration of how are they distributed.

As can be observed from the figure and the table, most of the simulated cases are auxetic i.e., $\nu_{t}<0$ since in the 75% quartile of its distribution, the Poisson’s ratio is lower or equal than 0. This is due to the distribution of $D_{joint}$—the main variable controlling the auxeticity—which presents negative values up to the third quartile, at least. In Table 3, the cell parameters and Young’s modulus of three extreme cases for the Poisson’s ratio (i.e., $\nu_{t}\in \left\{-1,0,0.5\right\}$) are displayed, as well as a representation of the tessellation of a few unit cells.

The previously highlighted influence of the parameter $D_{joint}$ in the auxeticity of the metamaterial can be seen from the schematic drawings of Table 3. However, this relation is not always straightforward, as Figure 8b suggests. Moreover, rather different configurations can yield the same Young’s modulus; see, e.g., $\nu_{t}=-1$ and $\nu_{t}=0.5$.

The calculation demonstrates the range of Young’s moduli and Poisson’s ratios that can be obtained in the metamaterial. Moreover, there is no simple correlation between the geometrical descriptors of the metamaterial and the mechanical properties that can be used for its design. The next section shows that even with a very simplistic machine learning nonlinear regression, the mechanical properties in the loading direction can be predicted from the geometrical parameters (features) of the unit cell. With the same volume (and approximate density) of the unit cell and an equal lattice radius, a wide range of mechanical properties (both Young’s modulus and Poisson’s ratio) can be designed through an inverse analysis and achieved with the proper features. In practice, this is done by varying only the connectivity of the lattices and the position of the internal nodes, without changing the global shape of the unit cell. This is a very important property in the design because it means that the shape of the component can be designed independently of the metamaterial design.

### 3.3. Determination of the Mechanical Properties through Inverse Analysis with a Simple ML Model

In the design procedure, we typically know the desired final mechanical properties and aim to determine the corresponding metamaterial design. To perform such designs through inverse analysis, the general nonlinear relations between the set of cell geometric parameters and the desired behavior are obtained using a simple machine learning (ML) algorithm. This ML model is trained using the numerical beam model described in the previous subsection, which is used to obtain the mechanical properties for a variety of geometrical combinations.

Designing a metamaterial with the desired mechanical properties and embedding a lattice architecture made of GAM in complex geometries requires simulating models with a higher computational cost. Surrogate ML models can simplify this process by establishing the relationship between geometrical features and the desired behavior. They can either accelerate the computation of the behavior of the metamaterial cell for a variety of geometrical parameters or solve a progression of unit cell parameters in the component to obtain the desired whole component behavior. In this section, we demonstrate that these types of ML surrogate models can be easily created for such tasks.

To this end, the full data analysis cycle was applied, namely, gathering and cleaning the available data (data wrangling, [51]), statistical exploration of the data, generation/modification of the features (feature engineering, [52]), and applying an ML model with further validation in a test set.

After noticing some patterns relating to the cleaned input variables and the output (mechanical) variables in terms of the correlation coefficient, either the Pearson’s correlation [53] (Chapter 5) or Spearman’s correlation [54] (Chapter 19), in exploratory analyses of the data, an ML model was run to validate the feasibility of developing a surrogate model. The objective of this step is not to develop a highly complex ML model, but to fit a more classical, explainable ML model to illustrate this point. To this end, the available variables are separated into features (inputs) and targets (outputs), as is typically done, in order to fit a multi-output regressor. Namely,

- Inputs. Geometrical parameters: *H*, $H_{star}$, $D_{star}$, $D_{joint}$.
- Outputs. Mechanical properties: $E_{z}$, $\nu_{t}$

Regarding the regressor model, a random forest (RF) algorithm was used [55]—due to its simplicity and interpretability—making use of the scikit-learn library from the Python programming language [56]. As a loss function for this model, the mean squared error (MSE) was used, which, denoting the label (output) as *y*, is expressed as:

(4) $$\mathrm{MSE}=\frac{1}{n}\sum_{i=1}^{n}{\left(y^{\left(i\right)}-{\hat{y}}^{\left(i\right)}\right)}^{2},$$

where *n* is the number of samples and $y^{\left(i\right)}$ and ${\hat{y}}^{\left(i\right)}$ are the i-th values of the true and predicted labels, respectively. Additionally, the coefficient of determination $R^{2}$ is used as the metric to assess the performance of the model in the test set. This coefficient is expressed as [57]:

(5) $$R^{2}=1-\frac{{\mathrm{SS}}_{\mathrm{res}}}{{\mathrm{SS}}_{\mathrm{tot}}}=1-\frac{\sum_{i}{\left(y^{\left(i\right)}-{\hat{y}}^{\left(i\right)}\right)}^{2}}{\sum_{i}{\left(y^{\left(i\right)}-\bar{y}\right)}^{2}},$$

where SS stands for sum of squares, and $\bar{y}$ is the mean of the ground-truth output variable. Note that ${\mathrm{SS}}_{\mathrm{tot}}$ is a representation of the variance of the output data.

From the (raw) dataset of 10,539 samples, cases with meaningless values such as negative Young’s modulus or Poisson’s ratio out of bounds, as well as repeated cases, were dropped. This resulted in a cleaned dataset of 6598 cases. A train/test split was then performed randomly, with an 80%/20% scheme. After properly scaling the features and targets by means of a classical standardization, the RF model was run.

**Figure 9:**
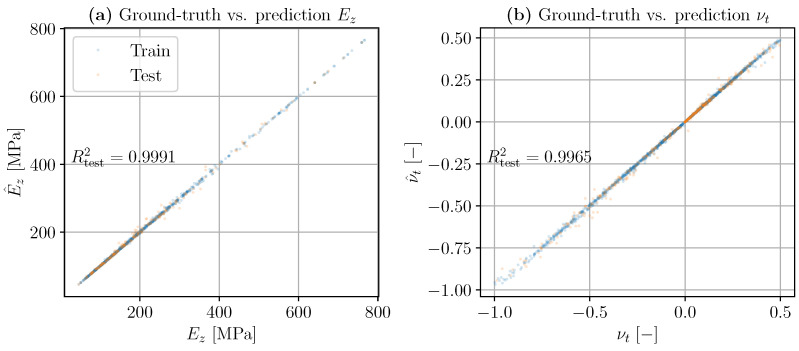
The ML model predictions of longitudinal Young’s modulus $E_{z}$ (**a**) and transverse Poisson’s ratio $\nu_{t}$ (**b**), using RF as the regressor. Blue represents the training set, and orange represents the test set. Ground-truth values (from simulations) are on the horizontal axes, while predicted values are on the vertical axes (perfect prediction lies on the bisectrix). A high accuracy is achieved in the test set in terms of the coefficient of determination $R^{2}$.

The predictions of the two mechanical properties of interest in this paper (Young’s modulus $E_{z}$ and transverse Poisson’s ratio $\nu_{t}$) for the train and test sets are shown in Figure 9. In both plots, the true values are displayed on the horizontal axis, while the predictions are represented on the vertical axis, denoted by a hat decoration $\left(\hat{\cdot }\right)$. The coefficient of determination $R^{2}$ is used as a metric to assess the performance of the model, and this value is also displayed in the figure.

As it can be observed, the model performs well in this simulated dataset as the coefficients of determination of both mechanical properties, i.e., $R^{2}\left(E_{z},{\hat{E}}_{z}\right)$ and $R^{2}\left(\nu_{t},{\hat{\nu }}_{t}\right)$, are greater than 0.995, achieving a virtually perfect prediction—almost all of the data points lie on the bisectrix of the graph. Note that these predictions were carried out using a rather simple ML model, which shows that this technology may be used to develop complex concurrent structure–metamaterial designs in which the metamaterial has functionally graded structures to meet complex overall structural behavior.

## 4. Conclusions

The proposed (and patented) metamaterial design can be tailored ad libitum to reproduce a wide range of mechanical properties, both Young’s modulus and Poisson’s ratio, in the loading direction. We have shown that there is a direct relationship between the variation of the connectivity of the lattices and the position of the internal nodes and the resulting mechanical properties of the metamaterial. This metamaterial can exhibit a wide spectrum of auxetic or non-auxetic behaviors and mechanical properties, such as stiffness. The main salient feature of this triangularization-based tunable metamaterial cell is that the dimensions and connectivity (boundary) remain the same regardless of the auxeticity, enabling the development of very simple functionally graded materials, including shells and other complex topologies, and allowing for concurrent structure-metamaterial optimization to obtain desired component-level properties. Furthermore, the geometrical parameters defining the metamaterial cell are few and simple, with a direct relationship to the desired mechanical behavior. Very simple machine learning models can be generated as surrogate models to facilitate parametric nonlinear inverse analyses for the mentioned concurrent two-level design. The metamaterial has versatile mechanical properties and can be reproduced and calculated even with simple methodologies, which are important for success in the industry.

## Figures and Tables

**Table 1:** Base material parameters used in the GAM code.

*E* [GPa]	ν
200	0.33

**Table 2:** Basic statistics of geometrical and mechanical variables of the numerical model, namely the mean, standard deviation (std), minimum, quartiles, and maximum values.

	Mean	Std	Min	25%	50%	75%	Max
$D_{joint}$	−0.38	0.38	−1.40	−0.60	−0.36	−0.12	1.2
$H_{star}$	−0.09	0.12	−0.70	−0.14	−0.06	−0.01	0.4
$D_{star}$	0.38	0.20	0.05	0.20	0.40	0.55	0.7
*H*	2.41	0.69	1.50	2.00	2.50	3.00	3.5
$E_{z}$ [MPa]	184.78	159.64	0.00	87.78	148.62	242.30	766.2
$\nu_{t}$	−0.18	0.28	−1.00	−0.33	−0.10	0.00	0.5

**Table 3:** Cell parameters, Young’s modulus, and schematic representation of three extreme cases for the transverse (to the *z*-axis) Poisson’s ratio i.e., $\nu_{t}\in \left\{-1,0,0.5\right\}$.

	$D_{\mathrm{joint}}$	$H_{\mathrm{star}}$	$D_{\mathrm{star}}$	*H*	$E_{z}$ [MPa]	Representation
$\nu_{t}=-1$	−0.8	−0.24	0.25	2.0	272.03	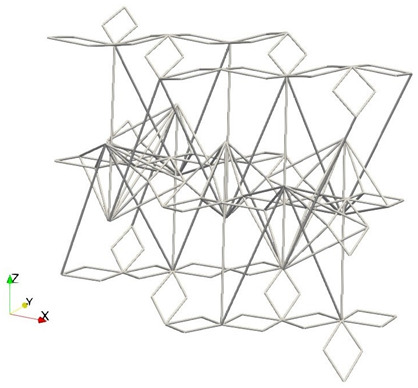
$\nu_{t}=0$	0.0	0.00	0.05	3.0	88.36	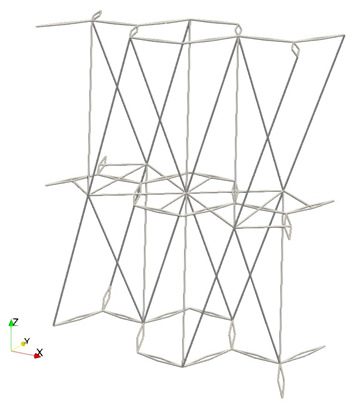
$\nu_{t}=0.5$	0.6	0.00	0.20	3.0	266.09	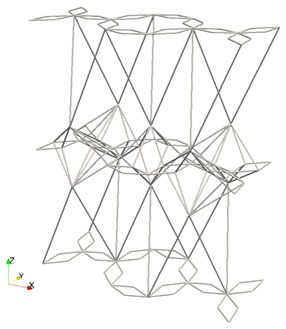

## Data Availability

For the data supporting, please contact the corresponding author.

## Appendix A. Structural Matrix Calculus Model and Equivalent Young’s Modulus and Poisson’s Ratio Calculation

The governing equation of the discretized structure in the static case is given by

(A1) Ku=f,

where K, u, and f are, respectively, the stiffness matrix, the nodal displacements, and the nodal force vectors of the whole structure. The stiffness matrices for both Euler–Bernoulli and Timoshenko with 12 degrees of freedom can be found in Equation (5.116) of [58]. Therefore, denoting the free degrees of freedom as *f* and the restricted ones as *r*, the previous system can be split as:

(A2) $$\left[\begin{array}{cc} K_{ff} & K_{fr}\\ K_{rf} & K_{rr} \end{array}\right]\left[\begin{array}{c} u_{f}\\ u_{r} \end{array}\right]=\left[\begin{array}{c} 0\\ f_{r} \end{array}\right],$$

where the nodal force vector in the free degrees of freedom is set to $f_{f}=0$ since the loading is purely displacement imposition. By performing static condensation, the vectors of nodal displacements of the free degrees of freedom $u_{f}$ can be obtained as follows:

(A3) $$u_{f}=-K_{ff}^{-1}K_{fr}u_{r},$$

and the nodal forces on the restricted nodes (reactions) are

(A4) $$f_{r}=\left(K_{rr}-K_{rf}K_{ff}^{-1}K_{fr}\right)u_{r}.$$

Then, for a uniaxial test, we prescribe a vertical displacement Δu at the top face nodes, while the vertical displacement at the bottom is restrained (e.g., $u_{r}$). The macroscopic imposed strain (continuum-equivalent) is given by:

(A5) $$\varepsilon_{zz}=\frac{\Delta u}{l_{z}},$$

where $l_{z}$ is the vertical dimension of the structure. To determine the strains in the two perpendicular directions, the macroscale displacements in the *x* and *y* directions are estimated by averaging the displacements at the external faces of the structure perpendicular to those directions. The strains $\varepsilon_{xx}$ and $\varepsilon_{yy}$ are then calculated by dividing the displacement magnitudes by their respective lengths. Once all strains are computed, Poisson’s ratios $\nu_{xz}$ and $\nu_{yz}$ are

(A6) $$\nu_{xz}=-\frac{\varepsilon_{xx}}{\varepsilon_{zz}},\;\nu_{yz}=-\frac{\varepsilon_{yy}}{\varepsilon_{zz}}.$$

Furthermore, by dividing the result of the reaction forces ***f***_*r*_ at the top face by the cross-sectional area perpendicular to the loading application, the stress $\sigma_{zz}$ can be estimated, and therefore the Young’s modulus can be computed as

(A7) $$E_{z}=\frac{\sigma_{zz}}{\varepsilon_{zz}}.$$

This model was validated by simulating the same structures using the Finite Element commercial software package, Nastran. Table A1 shows a comparison between the macroscopic results of the same test structure.

**Table A1:** Comparison of macroscale variable results between Nastran and the GAM code (code used for the ML examples).

	$\nu_{\mathrm{xz}}$	$\nu_{\mathrm{yz}}$	$E_{z}$ [MPa]
Nastran	−0.84	−0.73	174
GAM code	−0.85	−0.65	186

The results displayed in Table A1 are similar although not exact. This difference may be due to the beam model selected: Nastran uses the Timoshenko beam, whereas we implemented the Euler–Bernoulli beam.
